# Structural analysis of mitochondrial cytochrome *bc*_1_ complex with atovaquone bound reveals the molecular basis of antimalarial drug action

**DOI:** 10.1186/1475-2875-13-S1-P103

**Published:** 2014-09-22

**Authors:** Dominic Birth, Wei-chun Kao, Carola Hunte

**Affiliations:** 1 Institute for Biochemistry and Molecular Biology, ZMBZ, BIOSS Centre for Biological Signalling Studies, University of Freiburg, Freiburg im Breisgau, Germany; 2 Faculty of Biology, University of Freiburg, Freiburg im Breisgau, Germany

## Background

The substituted hydroxynaphthoquinone atovaquone is a potent antimalarial drug in use for prevention and therapy, a fundamental part of the ongoing global medicinal strategy to control the disastrous infectious disease. Atovaquone acts via inhibition of the cytochrome *bc*_1_ complex (cyt *bc*_1_) and related mutations were linked to parasitic drug-resistance.

## Materials and methods

The molecular binding mode of atovaquone was analysed by spectroscopy and X-ray crystallography. In addition, a comprehensive cytochrome *b* sequence analysis was performed to interpret binding interactions of the drug in context of sequence conservation of Qo site amino acid residues [1,2].

## Results

We determined the 3.0-Å resolution X-ray structure of mitochondrial cyt *bc*_1_ from *Saccharomyces cerevisiae* with atovaquone bound in the catalytic Qo site [1]. The drug, which has a pK_a_ of 6.9, forms a polarized H-bond between its ionized hydroxyl group and His181 of the Rieske protein subunit. Multiple non-polar interactions with side chains of cytochrome *b* residues stabilize hydroxynaphthoquinone and chlorophenyl-cyclohexyl groups. The cytochrome *b* sequence analysis showed that the majority of the interacting residues are conserved, so that atovaquone binding to the yeast cyt *bc*_1_ is likely to resemble the binding to the complexes of the target organisms.

## Conclusion

The binding mode provides detailed insights in the molecular basis of broad target spectrum, species-specific efficacies and acquired resistances. This may aid drug development to control the spread of drug-resistant parasites.
